# Long-Term Outcome after Rehabilitation of Bilateral Total Hip Arthroplasty in Renal Transplant Recipient – A Case Report

**DOI:** 10.3889/oamjms.2016.033

**Published:** 2016-02-25

**Authors:** Erieta Nikolikj Dimitrova, Aleksandar Adamov, Valentina Koevska, Biljana Mitrevska, Ivan Gacevikj, Arsim Agushi

**Affiliations:** 1 *Institute of Physical Medicine and Rehabilitation, Medical Faculty, Ss Cyril and Methodius University of Skopje, Skopje, Republic of Macedonia*; 2 *University Clinic for Orthopedic Surgery, Medical Faculty, Ss Cyril and Methodius University of Skopje, Skopje, Republic of Macedonia*

**Keywords:** renal transplantation, hip replacement surgery, rehabilitation, exercise, outcome

## Abstract

**INTRODUCTION:**

Total hip replacement is generally proposed for renal transplant patients with avascular osteonecrosis of the femoral head.

**PURPOSE:**

The purpose of the study is to report the long-term outcome after rehabilitation of bilateral total hip arthroplasty in a patient with renal transplantation suffering from avascular osteonecrosis of the both femoral heads.

**MATERIAL AND METHOD:**

The patient S.D, 49 years old at follow-up. Few months after renal transplantation, the patient had got avascular osteonecrosis of both femoral head. One year after transplantation the total hip arthroplasty for both hip joints were performed. Three years later repeat total hip arthroplasty surgery for left hip was performed. After any surgery intervention the patient was referred for inpatient rehabilitation. For clinical assessment the clinical findings and Harris Hip Score have been used. The rehabilitation program consisted of exercises, occupational therapy, and patient education.

**RESULTS:**

After any rehabilitation treatment the patient had improvement of clinical findings. At follow-up assessment outcome for both hip function was good - Harris Hip Score was 81 points.

**CONCLUSION:**

Rehabilitation is integral part of multidisciplinary treatment of renal transplant recipient after total hip arthroplasty. Regular exercise training of these patients is very important for improving of their long-term outcome.

## Introduction

Avascular osteonecrosis of the femoral head is a serious osseous complication after renal transplantation. It is caused by disruption of blood flow. Although the precise mechanism is still uncertain, the administration of glucocorticoid has been considered to play an important role in the occurrence of osteonecrosis. It prevalence clearly decreased from 20% to 4% after introduction of cyclosporine and reduction of steroid doses [1]. These patients have been proposed to be treated by elimination of weight–bearing, conservative treatment, but also they have been undergone core decompression and total hip replacement (THR) [2, 3].

This is our first patient with bone complication after renal transplantation who was admitted for inpatient rehabilitation treatment.

The purpose of the study is to report the long-term outcome (six years’ outcome) after rehabilitation of bilateral total hip arthroplasty in a patient with renal transplantation suffering from avascular osteonecrosis of the both femoral heads.

## Material and Method

The patient S.D, male, 49 years old at follow-up assessment, single, carpenter with 17 years working experience, with previously received disability pension due to chronic renal failure. Non smoker, he was previous smoker until six years ago. He has been undergone to renal transplantation on the right kidney because the chronic renal failure six years ago. His age at the time of the operation was 42 years. He had regular check in by nephrologists. Following surgery he has received immunosuppressive drugs, like cyclosporine and glucocorticoids to prevent rejection of the new organ.

**Figure 1:**
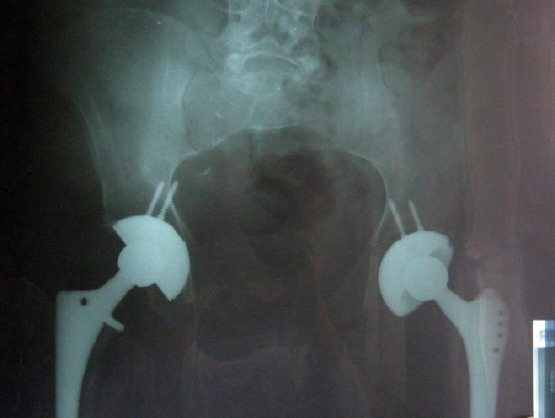
*X-ray of pelvis with both hips after both total hip artroplasty*.

Few months after renal transplantation, the patient had got hip pain due to avascular osteonecrosis of both femoral heads. The diagnosis was confirmed with native radiologic findings and magnetic resonance imaging (MRI) of the pelvis with both hips. In December 2008 the decompression of bilateral femoral head was performed. One year after transplantation (2009) a total hip arthroplasty (non- cemented) of the right hip and few months later a total hip arthroplasty (non- cemented) of the left hip were performed (Figure 1). Three years after first arthroplasty (2012) the patient has undergone the repeat total hip arthroplasty surgery for left hip due to prosthesis loosening. After any surgery intervention the patient was referred for inpatient rehabilitation in regional spa center and further rehabilitation in the rehabilitation hospital.

In January 2010 our patient has been suffering from low back pain and bilateral sciatica, and has been conservatively treated by orthopedic surgeon. The findings on computerized tomography (CT) scan of lumbar spine in the transversal plane confirmed lumbar disc herniation at L4-L5 level with compression of the cauda equina and nerve roots, with mild spinal canal stenosis. HLA typisation showed A2, A3, B18, B 27 positive findings. At the same time, dual energy x-ray absorptiometry (DXA) confirmed osteoporosis (t- score on right forearm -3.6, and L1-L4 -1.8), and bisphosphonate ibandronic acid 150 mg and vitamin D were administrated.

The patient was admitted for rehabilitation treatment six times, after total hip arthroplasty surgeries and also for exercise training and maintenance of his functional status.

At the first admission the patient also has suffered from low back pain, that reffers in left leg to the knee, pain in the left knee after long walking, limited range of motions in the both hips, weakness of the hip muscles, walking with the pair of below elbow crutches. The patient was obese, his height was 185 cm, weight was 100 kg, body mass index (BMI) 34.2. The patient’s cardiovasular system was well.

For clinical assessment the clinical findings, measurement of the both hip range of motion, leg length and leg circumference, hip muscle strength (manual muscle testing- MMT), Harris Hip Score [4] and Visual Analogue Scale (VAS) score for low back pain have been used. Assessments were made at baseline and at discharge during the all admissions, and at follow up 6 years after first admission.

The clinical findings of musculoskeletal system on the first admittion after both artroplasty surgery was: lumbar spine was slight scoliotic deviation in thoracolumbar spine, without spasm of lumbar spinal muscles, with reduced range of motions in the lumbar spine, anteflexion finger-floor distance 40 cm, reduced retroflexion. The right leg was shorter for 1 cm (umbilicus-maleol). There was hipotrophy of the tight muscles and hipotrophy of the calf muscles bilaterally, mostly peroneal muscles and slight hipotony of the below knee muscles on the left leg. There are scars from operaive cut on the outer part of the both hips. Active movements in the right hip were limited (especially flexion with extended knee and abduction because the muscle weakness (illiopsoas and gluteus medius MMT 4-), and in both knees and ankles were in normal range. Active movements in the left hip were limited with weakness of the hip flexor (MMT 3-) and abductor (MMT 4-), in the left knee were in normal range. Left foot was in slight equinovarus, dorsal flexion in the ankle was limited, especially eversion, but pasive dorsal flexion was 10°, plantar flexion was in normal range. There were muscle weakness of the dorsal flexion of the second, trird and forth finger on the left leg. There wasn’t any sensitive disturbance. Patelar reflex was symetrical, reflex of Achilles tendon was diminished on the left. He walked with the aid of below-elbow crutches. VAS score for low back pain at admission was 7.

**Figure 2:**
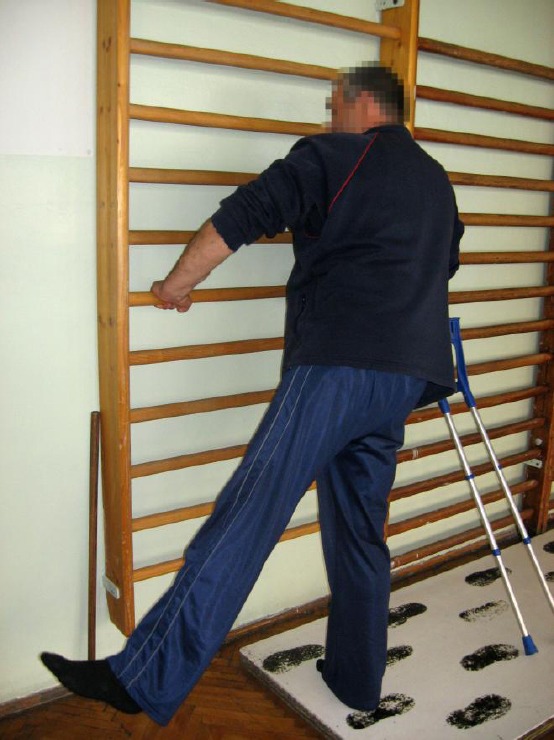
*Exercises for hip abductor*.

The rehabilitation program consisted of range of motion exercises, strengthening exercises for hip muscles (abductor, flexion, extensor muscles) (Figure 2), strengthening exercises for quadriceps, stationary bicycle exercises, isometric exercise for spinal, abdominal and gluteal muscles, occupational therapy and patient education about hip arthroplasty and ergonomic advices for spine biomechanics. For low back pain transcutaneous electrical nerve stimulation (TENS) and dyadinamic currents were applied.

During the rehabilitation he continued to receive immunosuppressive drugs like mycophenolate mofetil, cyclosporine, glucocorticoids; and also antihypertensive therapy, bisphpsphonate, statine, diuretic, folic acid, and acetilsalicil; and bisphosphonate therapy and vitamin D for osteoporosis.

## Results

After all inpatient rehabilitation treatment the patient had improvement in clinical findings (improved muscle strength, slightly increased range of motions in both hips, better ambulatory with one or both crutches, decreased low back pain).

At follow-up examination six years after bilateral replacement surgery and repeat hip arthroplasty of the left leg functional status of both hips was good with Harris Hip Score 81 points. The patient was obese and had mostly sedentary life-style without any physical activities. He hasn’t performed exercises at home regularly, he didn’t lose the weight. He drove his car. Clinically there was some muscle weakness of the hip muscles more on the left leg, and limited range of motions in the both hips (Table 1).

**Table 1:** Range of motions (in degrees) in both hips during the first, second, the last admission on rehabilitation treatment and at follow-up 6 years later

Active range of motion of both hips (in degrees) at admission (a) and at discharge(d)	Left leg I admission a/d	Left leg II admission a/d	Left leg Last admission a/d	Right leg I admission a/d	Right leg II admission a/d	Right leg Last admission a/d	Follow-up Six years later
Flexion with extended knee	0^0^/10^0^	50^0^/55^0^	50^0^/80^0^	65^0^/65^0^	20^0^/60^0^	80^0^/80^0^	10^0^ left 70^0^ right

Flexion with flexed knee	0^0^/30^0^	70^0^/75^0^	70^0^	90^0^	90^0^/95^0^	95^0^/N	20^0^ left 85^0^ right

Extension	-5^0^/0^0^	-5^0^/-5^0^	-10^0^/-5^0^	0^0^/5^0^	0^0^/5^0^	-10^0^/-5^0^	-10^0^ left -5^0^ right

Abduction	0^0^/10^0^	15^0^/20^0^	25^0^/35^0^	5^0^/10^0^	20^0^/25^0^	40^0^/40^0^	30^0^ left 35^0^ right

He has suffered periodically from slight low back pain. VAS score for low back pain was 2. His gait was with a pair of below elbow crutches. The follow-up x-ray of the pelvis with hips presents situation after total arthroplasty on both hips.

## Discussion

Bone diseases in kidney transplant recipients have been previously reported. This is our first patient with bone complication after renal transplantation who was admitted for inpatient rehabilitation treatment. Following renal transplantation he has permanently received immunosuppressive therapy which includes corticosteroids. Avascular osteonecrosis of the both femoral heads was developed during the first year after renal transplantation.

Avascular necrosis under immunosuppressive therapy is a well known sequel following solid organ transplantation. Most cases affect hip, knees or shoulders in more than one location and occur in conection with the use of high-dose steroids. In a case report was presented that despite the steroids had been completely withdrawn after avascular necrosis of the femoral head, 2 years after renal transplantation, MRI revealed bilateral tibial and tarsal bone necrosis [5]. Shibatani M. et al. investigated the development of avascular osteonecrosis after renal transplantation using MRI in 150 patients (96 males). They confirmed that the total dose of steroids given within the first 2 months after renal transplantation had a great influence on the incidence of avascular osteonecrosis [6].

In the retrospective study of 758 kidney transplant patients, for post-operative immunosupression, 374 patients had received high-dose corticosteroids (average 12.5 g during the first year post-operatively), and 376 patients had received low-dose corticosteroids (average 6.5 g during the first year post-operatively) and cyclosporine A. In high-dose steroid group 11.2% developed femoral head necrosis, and in the low-dose steroid group only 5.1% patients developed this complication at an average of 26.2 months and 20.5 months respectively post-transplantation [7].

In the retrospective study, among 305 renal transplant recipients 14 patients (4.5%) developed osteonecrosis of the femoral head, which was bilateral in 12 patients and unilateral in two. Thus, total number of hips with osteonecrosis was 26. The mean interval between transplantation and magnetic resonance imaging was 8.9 months. Extensive necrosis was found in most cases at the first evaluation (> 25% in 15 cases and > 50% in eight). Eleven patients were treated by elimination of weight-bearing and conservative treatment and 15 underwent core decompression. Mean follow-up was 33 months since transplantation. A poor functional outcome (Lequesne’s index > 7 or total hip arthroplasty) was seen in 61.5% of cases [2].

In the historical cohort study consisted of 42,096 renal transplant recipients enrolled in the United States Renal Data System between 1 July 1994 and 30 June 1998, renal transplant recipients had a cumulative incidence of total hip arthroplasty of 5.1 episodes/1000 person-years, which is 5-8 times higher than reported in the general population. Avascular necrosis of the hip was the most frequent primary diagnosis associated with total hip arthroplasty in this population (72% of cases). Repeat surgeries were performed in 27% of the patients with avascular necrosis, vs.15% with other diagnosis. Total hip arthroplasty is well tolerated and is not associated with increased mortality in this population [8].

One year after kidney transplantation in our patient the total hip arthroplasty for both hip joints were performed due to femoral head osteonecrosis. Three years later repeat total hip arthroplasty surgery for left hip due to prosthesis loosening was performed. After any surgery intervention the patient was referred for inpatient rehabilitation.

A review of the literature reveals that cemented hip arthroplasty provides good to excellent functional outcomes for renal transplant patients. Recent studies have found that despite decreased bone stock in these patients, porous-coated prostheses are not contraindicated [9].

During the inpatient rehabilitation treatment our patient performed strengthening exercises for hip muscles (abductor, flexion, and extensor muscles), quadriceps exercise, range of motion exercises, stationary bicycle exercises, isometric exercise for spinal, abdominal and gluteal muscles, exercises in open and closed chain, occupational therapy and education.

Total hip arthroplasty has improved the care of patients with end-stage joint disease, leading to pain relief, functional recovery, and substantial improvement in quality of life. However, long-term studies indicate persistence of impairment and functional limitation after total hip arthroplasty, and the optimal rehabilitation protocols are largely unknown. In the systematic review the convincing evidence for the effectiveness of single interventions in addition to usual exercise programs exists for each of the three following options: treadmill training with partial body-weight support, unilateral resistance training of the quadriceps muscle (operated site), and arm-interval exercises with an arm ergometer. In the late postoperative phase (operation interval > 8 weeks) exercise programs consistently improve both impairment and ability to function. Weight-bearing exercises with hip-abductor eccentric strengthening may be the crucial component of the late-phase protocols [10].

Despite a successful surgical procedure, deficits in muscle strength and physical function are documented 1-2 years after total hip replacement. There is a lack of evidence concerning which rehabilitation strategy is most effective after THR. In the single-blinded, cluster randomized controlled trial with consecutive sample of 46 patients undergoing primary THR surgery for osteoarthritis, 44 patients completed the trial. An intervention group received 12 weeks of intensified exercises (e.g. rubber band resistance) and a control group received standard rehabilitation exercises without external resistance. Hip abduction strength was significantly weaker in the leg operated compared with the leg not operated on after the intervention in both groups. The authors concluded that the majority of THR patients tolerated early-initiated intensified exercises without additional pain and with high patient satisfaction, but some of the patients need supervision to perform intensified exercises [11].

In the other clinical trial with 26 patients who had had a total hip arthroplasty the effect of home versus in-hospital exercise under supervision programs on hip strength, gait speed and cadence were evaluated. The best improvement in maximum isometric abduction torque was showed in the group which exercised under physiotherapy supervision in hospital [12].

Our patient made DXA examination two years after renal transplantation that demonstrated signs of osteoporosis, so bisphosponate medication and vitamin D were administrated.

Osteoporosis, osteopenia, and osteonecrosis are common in renal transplant recipients. In the clinical study of 85 renal transplant recipients with mean age of 36.25 ± 10.5 years and mean duration of posttransplantation follow-up of 9.82 ± 2.72 months; t scores of forearm and lumbar vertebrae were normal in 29.4% and 21.2%; osteopenia in 56.5% and 49.4%; and osteoporosis in 12.1% and 29.4% of patients, respectively. They concluded that bone disease including osteopenia and osteoporosis was observed among 70%. During the follow-up period, BMD increased significantly from baseline at 9.82 ± 2.72 months. VitD therapy caused more prominent improvements in BMD [13].

In the review of 24 RC trials (1.299 patients) was presented that fracture risk for a kidney transplant recipient is four times that of the general population and higher than for a patient on dialysis. Bisphosphonates (any route), vitamin D sterol, and calcitonin all had a beneficial effect on the bone mineral density at the lumbar spine. Bisphosphonates and vitamin D sterol also had a beneficial effect on the bone mineral density at the femoral neck. Bisphosphonates had greater efficacy for preventing bone mineral density loss when compared head-to-head with vitamin D sterols. The authors concluded that treatment with bisphosphonates, vitamin D sterol or calcitonin after kidney transplantation may protect against immunosuppression-induced reductions in bone mineral density and prevent fracture. Adequately powered trials are required to determine whether bisphosphonates are better than vitamin D sterols for fracture prevention in this population. The optimal route, timing, and duration of administration of these interventions remain unknown [14].

In the other systhematic review of RCTs conducted to assess the evidence avalible to guide targeted treatment to reduce bone disease in transplant recipients, the authors concluded that bisphosphonates and vitamin D have a beneficial effect on BMD at the lumbar spine and femoral neck [15]. After renal transplantation, both bisphosphonates and vitamin D metabolities, variably associated with calcium supplementation, have been demonstrated to have beneficial effect on bone loss, at least in the first year after renal transplantation. However, there are no data about the possible efficiacy of these treatments on fracture rate [16].

At follow-up assessment our patient has good postoperative outcome with Harris Hip Score of 81 points. Our opinion is that he should have better outcome if he had reduced his weight and if he performed the exercises regularly.

In conclusion, renal transplant recipient may develop avascular femoral head necrosis and osteopenia/osteoporosis due to corticosteroid therapy. Some of these patients need total hip arthroplasty. Rehabilitation is a integral part of multidisciplinary treatment of renal transplant recipient after total hip arthroplasty. Regular exercise training of these patients is very important for improving of their long-term outcome.

## References

[1] Hedri H, Cherif M, Zouaghi K, Abderrahim E, Goucha R, Ben Hamida F, Ben Abdallah T, Elyounsi F, Ben Moussa F, BenMaiz h, Kheder A (2007). Avascular osteonecrosis after renal transplantation. Transplant Proc.

[2] Le Parc JM, Andre T, Helenon O, Benoit J, Paolaggi JB, Kreis H (1996). Osteonecrosis of the hip in renal transplant percipients. Changes in functional status and magnetic resonance imaging findings over three years in three hundred five patients. Rev Rhum Engl Ed.

[3] Paydas S, Balal M, Demir E, Sertdemir Y, Erken U (2011). Avascular osteonecrosis and accompanying anemia, leucocytosis, and decreased bone mineral density in renal transplant recipients. Transplant Proc.

[4] Harris WH (1969). Traumatic arthritis of the hip after dislocation and acetabular fracture;treatment by mold arthroplasty. J Bone joint Surg.

[5] Vogel M, Starch K, Ehren K, Woitas R, Wasmuth JC (2010). Avascular nevrosis of the bone after organ transplantation. Internist (Berl).

[6] Shibatani M, Fujioka M, Arai Y, Takahashi K, Ueshima K, Okamoto M, Yoshimura N, Hirota Y, Fukushima W, Kubo T (2008). Degree of corticosteroid treatment within the first 2 months of renal transplantation has a strong influence on the incidence of osteonecrosis of the femoral head. Acta Orthop.

[7] Inoue S, Horii M, Asano T, Fujioka M, Ogura T, Shibatani M, Kim WC, Nakagawa M, Tanaka T, Hirota Y, Kubo T (2003). Risk factors for nontraumatic osteonecrosis of the femoral head after renal transplantation. J Orthop Sci.

[8] Bucci JR, Oglesby RJ, Agodoa LY, Abbott KC (2002). Hospitalizations for total hip arthroplasty after renal transplantation in the United States. Am J Transplant.

[9] Nowicki P, Chaudhary H (2007). Total hip replacement in renal transplant patients. J Bone joint Surg Br.

[10] Di Monaco M, Vallero F, Tappero R, Cavanna A (2009). Rehabilitation after total hip arthroplasty:a systematic review of controlled trials on physical exercise programs. Eur J Phys Rehabil Med.

[11] Mikkelsen LR, Mikkelsen SS, Christensen FB (2012). Early, intensified home-based exercise after total hip replacement--a pilot study. Physiother Res Int.

[12] Unlu E, Eksioglu E, Aydog E, Aydog ST, Atay G (2007). The effect of the exercise on hip muscle strength, gait speed and cadence in patients with total hip arthroplasty:a randomized controlled study. Clin Rehabil.

[13] Sikgenc MM, Paydas S, Balal M, Demir E, Kurt C, Sertdemir Y, Binokay F, Erken U (2010). Bone disease in renal transplantation and pleotropic effects of vitamin D therapy. Transplant Proc.

[14] Palmer SC, McGregor DO, Strippoli GF (2007). Interventions for preventing bone disease in kidney transplant recipients. Cochrane Database Syst Rev.

[15] Palmer SC, Strippoli GF, McGregor DO (2005). Interventions for preventing bone disease in kidney transplant recipients:a systematic review of randomized controlled trials. Am. J Kidney Dis.

[16] Messa P, Aroldi A, Villa M, Rusconi E (2004). Bone complications of renal transplantation. How to identify and prevent them. G Ital Nefrol.

